# Preservation of the Aortic Root During Type A Aortic Dissection Surgery: An Effective Strategy?

**DOI:** 10.1055/s-0041-1725074

**Published:** 2021-10-07

**Authors:** Simon Dang Van, Jihed Laribi, Frédéric Pinaud, Patrice Binuani, Serge Willoteaux, Christophe Baufreton, Olivier Fouquet

**Affiliations:** 1 Department of Cardiac Surgery, University Hospital of Angers, Angers, France; 2 Department of Radiology, University Hospital of Angers, Angers, France; 3 Mitovasc Institute INSERM U1083 UMR-CNRS 6214, University Hospital of Angers, Angers, France

**Keywords:** aortic dissection, aortic root, late reoperation, supracoronary aortic replacement

## Abstract

**Background** Management of the aortic root during acute Type A aortic dissection (TAAD) repair remains controversial in term of long-term evolution and reoperation. The aim of this study was to assess the long-term outcomes of the aortic root after conservative management during primary surgery.

**Methods** One hundred sixty-four consecutive patients were included in this monocentric retrospective study. The primary endpoint was reoperation on the aortic root during long-term follow-up. Forty-six patients had aortic root replacement (ARR) and 118 had supracoronary aortic replacement (SCR). The 10-year survival, occurrence of significant aortic regurgitation, and radiologic aortic root dilatation in each group were assessed during follow-up.

**Results** Patients from ARR group were younger than those from SCR group ( *p* < 0.0001). Median follow-ups of ARR group and SCR group are 4.4 (interquartile range [IR]: 2.6–8.3) and 6.15 (IR: 2.8–10.53) years, respectively. Reoperation of the aortic root during long-term follow-up was similar in both groups (ARR group: 5.1%, SCR group: 3.3%, *p* = 0.636). The 10-year survivals of ARR and SCR groups were 64.8 ± 12.3% and 46.3 ± 5.8% ( *p* = 0.012), respectively. Long-term significant aortic regurgitation occurred in one patient (1.7%) and seven patients (7.6%) of the ARR and SCR groups ( *p* = 0.176), respectively. Radiologic aortic root diameters in the SCR group were similar between postoperative period and follow-up studies ( *p* = 0.58). Reoperation on the distal aorta ( *p* = 0.012) and patent radiologic false lumen of the descending aorta ( *p* = 0.043) were independent risk factors of late death.

**Conclusion** SCR is an effective technique for primary TAAD surgery and does not increase the rate of late reoperation on the aortic root.

## Introduction

Acute Type A aortic dissection (TAAD) remains a catastrophic disease with a high rate of mortality that requires emergency surgery. One of the most complex aspects of this surgery is to determine the extent of the resected ascending aorta. A conservative approach with supracoronary replacement (SCR) of the ascending aorta and resuspension of the aortic valve is a widespread technique that provides acceptable early mortality rate. 1 However, preserving the aortic root during surgery for TAAD may expose to several concerns during long-term follow-up. Indeed, recurrence of aortic regurgitation, secondary aortic root aneurysm, proximal false lumen aneurysm, and recurrent dissection of the aortic root are described unfavorable outcomes after TAAD surgery treated with a conservative technique. 2 Replacement of the aortic root (such as Bentall, Tirone David, or Yacoub procedures), may prevent late reoperation on the aortic root through their total resection of the pathologic aortic wall, including sinus of Valsalva. 3 4 On the other hand, such techniques are more complex than SCR and may increase perioperative morbidity and mortality. 5 A few studies suggest that a more extensive approach to the aortic root during TAAD surgery provides excellent outcome and decreases aortic root complications and late reoperation. 4 6 However, others studies 7 do not show increased early and late mortality for proximal reoperation of the aortic root after a previous TAAD conservative surgery on the aortic root.

The aim of this study was to compare the long-term results and outcomes of aortic root replacement (ARR) versus supracoronary replacement during TAAD repair.

## Materials and Methods

### Population

Between January 1990 and December 2014, 164 consecutive patients underwent aortic surgery for TAAD at the University Hospital of Angers. A patient database was constituted retrospectively using clinical records, imaging, and operative reports. Patients were divided into following two groups according to the operative management of the aortic root during TAAD surgery: (1) SCR of the ascending aorta and (2) ARR. Individual consent and institutional review board approval were waived because they were not required by the ethics committee according to French regulation, concerning this retrospective observational study using data collected from clinical patient's records only.

### Surgical Technique

According to surgeon's discretion, arterial cannulation sites were the femoral or the axillary arteries. Venous drainage was ensured by simple or double venous cannulation (superior and inferior vena cava, femoral vein and superior vena cava, or a simple right atrial cannulation). Cerebral protection during circulatory arrest for open arch surgery was performed with moderate (25–28°C) or deep hypothermia (18–20°C). Retrograde cerebral perfusion through the superior vena cava 8 was mostly performed until 2010. Antegrade cerebral perfusion through the right axillary artery, the left common carotid, and/or the right brachiocephalic artery was the preferred strategy of cerebral perfusion since 2010. Cerebral perfusion flow rate was 10 mL/kg/min. No visceral perfusion was performed during circulatory arrest. If needed, the proximal and/or distal residual aortic wall was repaired with surgical glue (Biolgue, Cryolife, Kennesaw, GA).

In case of nonconservative surgery of the aortic root, modified Bentall procedure or remodeling or inclusion technique of the aortic root described by David and Feindel 9 and Sarsam and Yacoub 10 was performed.

### Postoperative Management

Postoperative outcomes were retrospectively collected from the patient's clinical file. An aortic computed tomography or magnetic resonance imaging and a transthoracic echocardiography were performed at discharge from hospital.

### Follow-up

Clinical and radiological follow-ups were performed for all patients alive at discharge from hospital. Data were collected from the patient's clinical record. Data from patients lost to direct follow-up were collected by telephone interview from their attending physicians or cardiologists. Radiographic analysis of the aortic imaging was performed by standardized measures of the aortic root, ascending aorta, aortic arch, and descending aorta and compared between the postoperative and follow-up periods. Patency of the residual false lumen and perfusion deficits were also analyzed. Patency of the false lumen was divided into two groups as follows: patent false lumen or a partially thrombosed lumen versus a completely thrombosed false lumen. All measures were obtained after two-dimensional (2D) and three-dimensional (3D) image reconstruction with Osirix software (Pixmeo SARL, Bernex, Switzerland) employing orthogonal views of the different segments of the aorta to provide reliable diameters.

### Endpoints

The primary endpoint was the occurrence of reoperation of the aortic root. Secondary endpoints were death during hospitalization and follow-up, occurrence of aortic regurgitation ≥ grade 2, reoperation of the distal aorta, and aortic root dilatation (defined as a maximal diameter at the sinuses of Valsalva in relation to body surface area higher than 21 mm/m ^2^ 11 ). In-hospital mortality was defined as the occurrence of death within 30 days after surgery. Patients dead within these 30 days were excluded from the long-term analysis. Postoperative and late radiologic diameters of aortic root were compared between groups.

### Statistical Analysis

Statistical analysis was performed using SPSS Statistics software (IBM Corporation, New York, NY). Categorical variables were expressed as percentages. Continuous variables were reported as mean ± standard deviation (SD). Continuous variables were compared between groups using *t* -test or Mann–Whitney *U* -test when nonnormally distributed, whereas categorical variables were compared using the Chi-square test or the Fisher's exact test when appropriate. Event-free survival curves were calculated using the Kaplan–Meier method. Survival curves were compared using the log-rank test. The influence of the type of surgery on postoperative death and freedom from aortic root pejorative evolution was assessed by univariate and multivariate Cox analyses, adjusted on confounding factors. A *p* -value of <0.05 was used for statistical significance.

## Results

### Preoperative Characteristics

One hundred and sixty-four patients were included in the study. Patient's baseline characteristics are summarized in Table 1 . Mean age of the entire cohort was 61.12 ± 14.1 years. Forty-six patients and 118 patients underwent ARR and SCR, respectively. Patients from the ARR group were younger (ARR group: 51.65 ± 16.35, SCR group: 64.81 ± 11.17, *p* < 0 0.0001), had more annuloaortic ectasia disease (ARR group: 20.5%, SCR group 0.9%, *p* < 0.0001) and had more significant aortic regurgitation ≥ 2 (ARR group: 65.9%, SCR group 44.4%, *p* = 0.016) than patients from the SRC group. Preoperative data imaging ( Table 2 ) showed more primary intimal tear in the sinus of Valsalva in the ARR group than in the SCR group (ARR group: 43.2%, SCR group: 13.6%, *p* < 0.0001) and more patent false lumen in the sinus of Valsalva (ARR group: 86.8%, SCR group: 56%, *p* = 0.001).

**Table 1:** Population baseline characteristics

	SCR group ( *n* = 118)		ARR group ( *n* = 46)		*p* -Value
	*n*	%	*n*	%	
Male gender	71	60.2	40	87	**0.001**
Age (y)	64.81 ± 11.17		51.65 ± 16.35		**<0.0001**
*Cardiovascular risk factors* :					
Hypertension	88	77.2	24	54.5	**0.006**
Smoking	32	28.6	12	27.9	0.935
Obesity	31	27	5	11.4	**0.036**
Dyslipidemia	37	32.2	4	9.1	**0.002**
Diabetes	12	10.4	4	9.1	1
Heredity of aortic disease	7	6.1	10	22.7	**0.007**
Height (cm)	167.34 ± 8.45		174.11 ± 9.04		< **0.0001**
Weight (kg)	78.17 ± 18.14		76.78 ± 12.43		0.637
Body surface area (m ^2^ )	1.85 ± 0.30		1.90 ± 0.17		0.341
Marfan disease	2	1.7	4	9.1	0.05
Bicuspid aortic valve	3	2.7	5	11.4	**0.043**
Annuloaortic ectasia	1	0.9	9	20.5	**<0.0001**
Pregnancy	0	0	1	2.2	0.278
Iatrogenic dissection	4	3.4	0	0	0.576
*Medical history of* :					
Obliterant arteriopathy	9	7.8	1	2.3	0.286
Chronic renal failure	2	1.7	3	6.8	0.130
Obstructive chronic bronchitis	6	5.2	1	2.3	0.674
Gastroduodenal ulcer	4	3.5	1	2.3	1
Cancer	7	6.1	6.1	0	0.191
Myocardial infarction	5	4.3	0	0	0.323
*Previous cardiac surgery* :	5	4.3	2	4.4	1
Aortic valve surgery	4	3.4	1	2.3	1
Aortic surgery	1	0.9	1	2.3	1
NYHA status ≥2	10	8.5	5	11.1	0.560
Preoperative shock/tamponade	29	24.6	10	21.7	0.701
Mesenteric ischemia	6	5.1	0	0	0.188
Limb ischemia	15	12.7	4	8.9	0.497
Acute aortic dissection	110	93.2	45	97.8	0.447
LVEF (%)	56.97 ± 14.49		61.32 ± 3.94		0.031
Aortic regurgitation ≥2	48	44.4	29	65.9	**0.016**
Pericardial effusion	48	44.4	20	48.8	0.635

Abbreviations: ARR, aortic root replacement; LVEF, left ventricular ejection fraction; NYHA, New York Heart Association; SCR, supracoronary aortic replacement.

**Table 2:** Preoperative imaging data

	SCR group ( *n* = 118)		ARR group ( *n* = 46)		*p* -Value
	*n*	%	*n*	%	
Bicarotid trunk artery	18	21.2	10	26.3	0.530
*Primary intimal tear location:*					
Sinus of Valsalva	15	13.6	19	43.2	< **0.0001**
Ascending aorta	67	60.9	23	52.3	0.433
Aortic arch	19	17.3	2	4.5	0.065
Descending aorta	9	8.2	0	0	0.062
*Aortic dissection involving* :					
Sinus of Valsalva	67	69.1	34	89.5	0.140
Aortic arch	83	85.6	34	89.5	0.548
Descending aorta	63	65.6	26	68.4	0.757
Abdominal aorta	56	58.3	21	56.8	0.869
*Patent false lumen* :					
Sinus of Valsalva	51	56	33	86.8	**0.001**
Aortic arch	66	70.2	31	81.6	0.180
Descending aorta	52	55.9	26	68.4	0.186
Abdominal aorta	48	51.6	21	56.8	0.596

Abbreviations: ARR, aortic root replacement; SCR: supracoronary replacement.

Note: 59 patients (35.9%) of 164 patients of the cohort presented a DeBakey Type 2 dissections.

### Operative Data

Operative data are summarized in Table 3 . Between 1990 and 2000, 37 patients underwent surgery for TAAD in our institution and 8 patients (21.6%) of them had ARR with the Bentall technique during this period. Between 2001 and 2014, 127 patients were operated for TAAD and 38 patients (29.9%) of them had ARR with the modified Bentall technique, Yacoub technique, and Tirone David technique. Surgical observation of the dissected structures showed more dissected sinus of Valsalva in the ARR group than in the SCR group (SCR group: 1.24 ± 1.20, ARR group: 1.80 ± 1.22, *p* = 0.009). In the ARR group, three patients (6.5%) had a Yacoub procedure, 19 patients (41.3%) had the Tirone David procedure and 24 patients (52.2%) had the Bentall procedure. Of the 24 patients, 10 patients (21.8%) had a biological aortic valve prosthesis and 14 (30.4%) had a mechanical aortic valve prosthesis. Ten patients of the SCR group (8.4%) underwent aortic valve replacement (AVR). Cardiopulmonary bypass time and cross clamp time were significantly longer in ARR group ( *p* = 0.002 and <0.0001, respectively). Eighty-four patients (71.2%) of the SCR group had additional glue used to reinforce the aortic root. Of them, 61 patients (72.6%) had Bioglue (Bioglue Surgical Adhesive, Cryolife Inc., Kennesaw, GA), 22 patients (26.2%) had Tissucol Kit (Baxter, Guyancourt 78280, France), and 1 patient (1.2%) had Fibrogel. No difference was observed in terms of reoperation on the aortic root during follow-up with or without previous use of biological glue in the SCR group ( *p* = 0.497). There was no difference concerning the management of the distal anastomosis between the two groups. Hemiarch replacement was the most performed technique in the management of the distal anastomosis in both groups. Antegrade cerebral perfusion was more frequently used in the ARR group than in the SCR group ( *p* = 0.029).

**Table 3:** Intraoperative data

	SCR group ( *n* = 118)		ARR group ( *n* = 46)		*p* -Value
	*n*	%	*n*	%	
Cardiopulmonary bypass time (min)	232.49 ± 87.11		279.28 ± 75.79		**0.002**
Cross-clamping time (min)	123.30 ± 49.77		191.91 ± 47.33		**<0.0001**
*Circulatory arrest* :					
Duration of circulatory arrest (min)	53.75 ± 30.99		40.57 ± 25.65		**0.026**
Temperature of circulatory arrest (°C)	20.71 ± 3.98		23.39 ± 4.95		**0.001**
*Cerebral protection during circulatory arrest* :					
Antegrade perfusion	29	24.8	19	42.2	**0.029**
Retrograde perfusion	66	56.4	15	32.6	**0.006**
Deep hypothermia	10	8.5	2	4.3	0.362
Number of dissected sinus of Valsalva	1.24 ± 1.20		1.80 ± 1.22		**0.009**
*Dissected aortic root structures* :					
Left coronary sinus	36	31.3	25	54.3	**0.006**
Right coronary sinus	31	67.4	47	40.9	**0.002**
Non coronary sinus	63	54.8	27	58.7	0.651
Left coronary artery	4	3.5	10	21.7	**<0.0001**
Right coronary artery	13	11.3	15	32.6	**0.001**
Use of glue in the aortic root	84	71.2	0	0	**<0.0001**
*Management of distal anastomosis* :					
Isolated supracoronary graft	52	67.5	25	54.3	0.236
Hemiarch replacement	53	44.9	16	34.8	0.237
Total arch replacement	5	4.2	3	6.5	0.687
Elephant trunk	8	6.8	2	4.3	0.727
Distal aortic gluing	60	50.8	20	43.5	0.396
*Aortic valve replacement* :					
Biological prosthesis	3	2.5	10	21.7	**<0.0001**
Mechanical prosthesis	7	5.9	14	30.4	**<0.0001**
Coronary arteries bypass graft	5	4.2	1	2.2	1
Thoracic endovascular aortic repair	3	2.5	0	0	0.560

Abbreviations: ARR, aortic root replacement; CABG, coronary arteries bypass graft; SCR, supracoronary replacement.

### Postoperative Outcomes

Postoperative data are summarized in Table 4 . There was no difference observed between the two groups concerning the in-hospital mortality ( *p* = 0.091), the intensive care unit length of stay ( *p* = 0.230), the total length of stay ( *p* = 0.262), pejorative neurological event, and reoperation for major bleeding ( *p* = 0.684). Less postoperative renal failure was observed in the ARR group ( *p* = 0.001). There was no difference between the two groups concerning early postoperative aortic regurgitation ≥ grade 2 ( *p* = 0.435).

**Table 4:** Postoperative clinical outcomes

	SCR group ( *n* = 118)		ARR group ( *n* = 46)		*p* -Value
	*n*	%	*n*	%	
In-hospital 30-day mortality	27	22.9	5	11.1	0.091
Total length of stay (d)	10 (7–18)		11 (10–16)		0.998
Intensive care unit length of stay (d)	5 (2–9)		4 (3–8)		0.589
Reoperation for major bleeding	5	4.7	3	7.5	0.684
Mechanical ventilation duration (d)	5.85 ± 11.08		3.25 ± 5.49		0.160
*Neurological outcomes* :					
Spinal cord ischemia	3	2.7	2	4.5	0.620
Stroke	10	9.1	2	4.5	0.512
Coma	6	5.5	0	0	0.186
Renal failure	60	57.1	11	27.5	**0.001**
Mediastinitis	4	3.8	0	0	0.576
Pulmonary infection	15	14.4	6	15	0.930
Prolonged mechanical ventilation (>3 days)	32	31.1	9	22.5	0.309
Acute respiratory distress syndrome (PaO _2_ /FiO _2_ < 200)	12	11.5	4	10	1
Confusion	28	26.9	6	15	0.131
Mesenteric ischemia/infarction	12	11.7	1	2.5	0.111
Left ventricular ejection fraction (%)	60.86 ± 6.48		60.26 ± 6.65		0.643
Postoperative aortic regurgitation ≥2	6	7.6	1	2.9	0.435

Abbreviations: ARR, aortic root replacement; LVEF, left ventricular ejection fraction; SCR, supracommissural replacement.

Note: Total length of stay and intensive care unit length of stay are expressed in median and interquartile ranges (25–75%).

### Reoperation of the Aortic Root

**Fig. 1:**
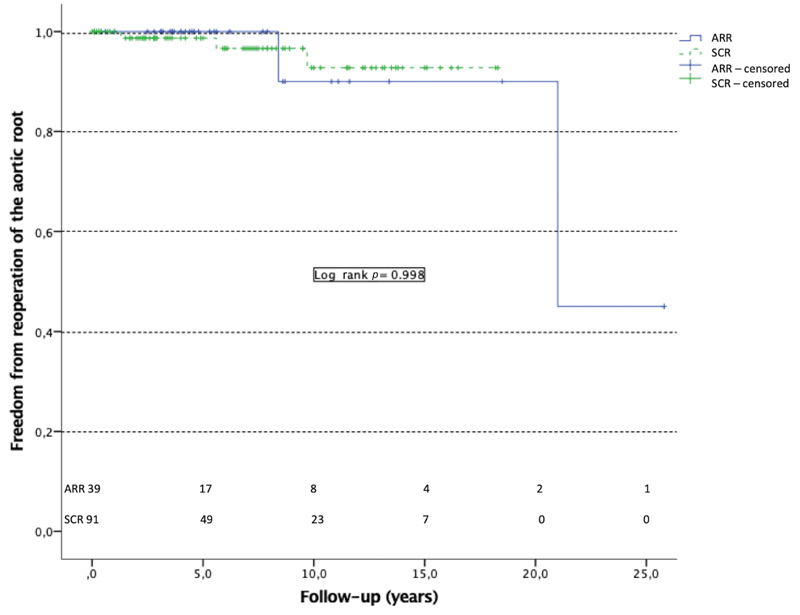
Freedom from reoperation of the aortic root. Occurrence of reoperation of the aortic root during follow-up was assessed in patients alive at discharge from hospital after primary operation. ARR, aortic root surgery; SCR, supracoronary replacement.

Freedom from reoperation on the aortic root period is summarized in Fig. 1 . One patient of the ARR group was lost to follow-up. Two patients (4.8%) from the ARR group were reoperated on the aortic root, one patient underwent aortic valve replacement 21 years after primary surgery for aortic stenosis of the biological prosthesis and another patient had aortic endocarditis on a biological prosthesis requiring aortic valve replacement. Both patients were alive at the end of follow-up without significant aortic regurgitation. Three patients underwent reoperation on the aortic root in the SCR group (3.3%), two patients (2.2%) presented pseudo aneurysm of the proximal anastomosis and one (1.1%) patient presented pseudoaneurysm of the right coronary artery. The two patients with proximal anastomosis aneurysm died after the reoperation from multiorgan failure. The patient with the right coronary anastomosis aneurysm was reoperated successfully 6 years after primary surgery. He died 3 years after reoperation because of a recurrent distal aortic anastomosis aneurysm compressing the upper airways. Mean periods before reoperation in the ARR group and SCR group were 14.7 ± 6.3 and 5.53 ± 2.42 years ( *p* = 0.400), respectively. There was no difference between the two groups concerning reoperation of the aortic root during long-term follow-up ( *p* = 0.636).

### Survival Analysis

**Fig. 2:**
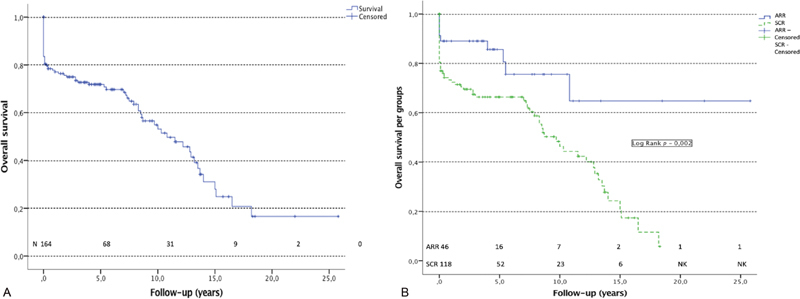
Kaplan–Meier analysis of the survival. ( **A** ) Overall survival, ( **B** ) overall survival per group. Overall survival analysis includes patients who died within 30 days after surgery and patients alive at discharge from hospital. ARR, aortic root replacement; SCR, supracoronary replacement.

Overall survival is shown in Fig. 2A . Data from 132 patients were available during follow-up. Mean survival was 11.10 ± 0.97 years for the entire cohort. Overall survival at 1, 5, and 10 years was 77.1 ± 3.3%, 70.8 ± 3.7%, and 53.2 ± 5.1%, respectively. Survival of each group is shown in Fig. 2B . The 5-year survival was 80.6 ± 5.6% in the ARR group and 66.4 ± 4.5% in the SCR group ( *p* = 0.012). The 10-year survival was 64 ± 12% and 46 ± 5.8% ( *p* = 0.012), respectively, in the ARR and SCR groups. Median follow-up of the ARR and the SCR groups is 4.4 (interquartile range [IR]: 2.6–8.3) and 6.15 (IR: 2.8–10.53; *p* = 0.241) years, respectively. Mean survivals for ARR and SCR groups were 18.56 ± 2.23 and 8.77 ± 0.69 years ( *p* = 0.002), respectively.

### Aortic Regurgitation ≥Grade 2

**Fig. 3:**
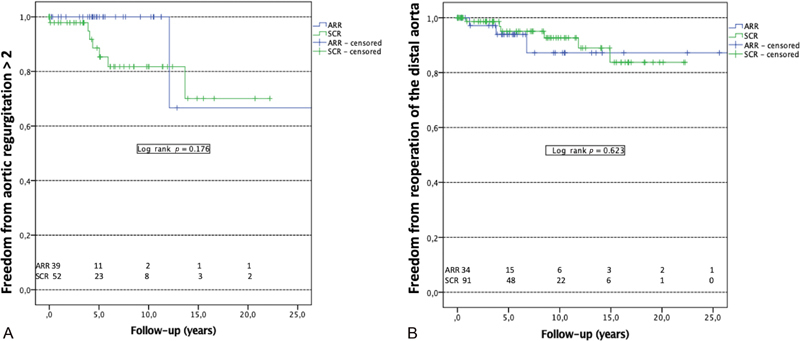
Aortic regurgitation ≥ grade 2 and reoperation of the distal aorta during follow-up. Occurrence during follow-up of significant aortic regurgitation ≥ grade 2 ( **A** ) and reoperation on the distal aorta ( **B** ) is analyzed in patients alive after primary surgery of Type A aortic dissection (patients who died within the 30 days after surgery are excluded from the present analysis). ARR, aortic root replacement; SCR: supracoronary replacement.

Freedom from new-onset aortic regurgitation ≥2 is shown in Fig. 3A . Data were available for 34 (82.9%) patients of the ARR group and 52 (57.1%) patients of the SCR group (patients lost to follow-up: ARR group, 7, SCR group, 39). One patient (1.7%) of the ARR (Yacoub technique) group had significant aortic regurgitation 9.9 years after primary surgery. Seven patients (7.7%) of the SCR group had significant aortic regurgitation during follow-up within a median period of 3.2 years (IR: 0–4.1 years). Occurrence of a significant aortic regurgitation ≥2 during follow-up was similar in both groups ( *p* = 0.176).

### Reoperation of the Distal Aorta

Freedom from distal aortic reoperation is shown in Fig. 3B . Distal aortic reoperation includes redo open surgery of the distal aorta and thoracic endovascular aortic repair (TEVAR). Mean period before reoperation of the distal aorta in ARR group and SCR group was 3.23 ± 2.3 and 4.2 ± 4.13 years ( *p* = 0.166), respectively. Three patients of the ARR group underwent open surgery of the aortic arch. Six patients of the SCR group underwent distal open arch reoperation and four patients had thoracic endovascular aortic endoprosthesis. There was no difference during follow-up concerning the reoperation of the distal aorta between the two groups ( *p* = 0.623).

### Risk Factors of Late Mortality

Univariate analysis was performed with patients alive at hospital discharge (ARR: *n* = 41, SCR: *n* = 91) after primary surgery. Significant preoperative aortic regurgitation ≥grade 2 ( *p* = 0.017), aortic valve replacement ( *p* = 0.031), postoperative radiologic descending aorta dissection ( *p* = 0.031), postoperative radiologic patent false lumen of the aortic arch and the descending aorta ( *p* = 0.029, *p* = 0.038), SCR ( *p* = 0.011), reoperation of the aortic arch ( *p* = 0.007), and postoperative pulmonary infection ( *p* = 0.005) were identified as significant risk factors for late death. Reoperation of the aortic root, recurrent aortic regurgitation ≥grade 2, and male gender were not a risk factor for late death ( *p* = 0.78, 0.084, and 0.344). Multivariate Cox analysis revealed following two variables as independent factors of late death: (1) reoperation of the aortic arch ( *p* = 0.012, 95% confidence interval [CI]: 1.286–7.761) and (2) postoperative radiologic patent false lumen of the descending aorta ( *p* = 0.043, 95% CI: 1.029–6.811). Reoperation of the aortic root ( *p* = 0.189, 95% CI: 0.732–11.472), recurrent significant aortic regurgitation ≥grade 2 ( *p* = 0.388, 95% CI: 0.209–1.838), and SCR ( *p* = 0.09, 95% CI: 0.846–10.257) were not independent predictors of late mortality.

### Radiologic Aortic Diameters Evolution

**Fig. 4:**
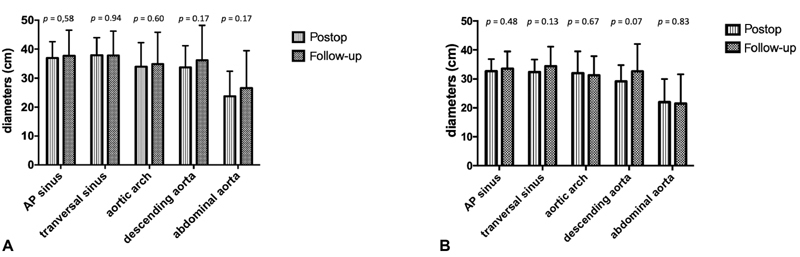
Evolution of radiologic aortic diameters at end of follow-up. ( **A** ) SCR group analysis, ( **B** ) ARR group analysis. Aortic radiologic diameters were compared in different sections of the aorta between early postoperative imaging and follow-up imaging. AP sinus: anteroposterior sinus diameter; ARR: aortic root replacement; Post-op, postoperative; SCR, supracoronary replacement.

Postoperative imaging data were available for 62 (52.5%) patients of the SCR group and 35 (76.1%) patients of the ARR group. End-of-follow-up imaging data were available for 56 (47.5%) patients of the SCR group and 34 (73.9%) patients of the ARR group. Postoperative and end-of-follow-up radiologic diameters of every different aortic section were compared in each group. Mean and median periods of radiologic follow-up in the SCR group were 4.3 ± 3.8 and 3.35 (IR: 0.6–11.1) years, respectively. Mean and median periods of follow-up in the ARR group were 3.69 ± 2.78 and 3.8 (IR: 0.8–6) years, respectively. Results are summarized in Fig. 4A and B . In both groups, no difference was observed between the postoperative imaging data and end-of-follow-up imaging data, especially concerning the evolution of the sinus of Valsalva diameters in the SCR group. Considering the maximal diameter indexed to the body surface area, 11 33 patients presented with aneurysms of the sinus of Valsalva during follow-up, defined as greater than 21 mm/m ^2^ (ARR group: 6 patients, SCR group: 27 patients, *p* = 0.003). Among these patients, four had significant aortic regurgitation and belonged to the SCR group.

## Discussion

The primary aim of surgery of TAAD is to prevent aortic rupture with catastrophic bleeding by resecting the primary aortic intimal tear, to correct aortic regurgitation if necessary, and to reestablish a dominant flow in the distal true lumen. Choosing the appropriate surgical procedure remains a surgical dilemma, for both proximal and distal aorta, considering the extent of the aortic dissection, the patient's comorbidities, and the risk of late reoperation. Operative strategies have changed over the decades, with different management policies for the aortic root and the aortic arch. Parikh et al 1 reports in the International Registry of Acute Aortic Dissection (IRAD) trends toward more extensive surgery of the proximal and distal aorta, using more valve-sparing procedures and fewer mechanical aortic prostheses. SCR with valve resuspension is considered as a relatively safe procedure to minimize the perioperative risk of pejorative outcomes. 12 13 On the other hand, some studies promote 14 an extensive management of the aortic root during TAAD surgery, primarily to avoid reoperation on the aortic root, with acceptable in-hospital and long-term mortality and low rate of reoperation. But most of the studies reported ARR in relatively young patients below 60 years of age, 4 15 while the mean age of occurrence of aortic dissection recently reported by Berretta et al 16 in the IRAD is 62 years. In our study, mean age of ARR group and SCR were significantly different, and may be the cause of most of the late differences observed between the two groups. ARR technique was also chosen if the aortic root presented structural abnormalities (aortic valve disease, primary intimal tear, and aortic wall defect) as suggested in the guidelines, 17 regardless of patient's age.

Rate of reoperation on the aortic root in our study was low and was not different between the ARR and SCR groups. In a meta-analysis of 19 studies, comparing conservative versus non conservative management of the aortic root, Saczkowski et al 18 reported no difference between the two strategies regarding the late mortality. Moreover, freedom from reoperation on the aortic root and recurrence of significant aortic regurgitation ≥grade 2 at 5 and 10 years were 89 and 79 and 95 and 86%, respectively. These results suggested that SCR was a safe procedure with an acceptably low rate of root complications and root reoperations at 10-year follow-up, considering the initial surgical issues during primary surgery. ARR technique was identified by some authors 14 as a protective factor against proximal reoperation, with improved long-term survival, avoiding the long-term problems of the SCR technique. 2 19 Reoperation on the aortic root after primary TAAD surgery remains risky, with early mortality of 11.1 to 14.3%. 5 7

However, extensive management of the aortic root during primary surgery, by consensus, should be performed in cases of aortic dissection with underlying connective tissue disorder like Marfan syndrome, 20 and should be considered in young patients. 15

Concerning radiologic diameter evolution of the aortic root after TAAD surgery, only a few studies analyzed and compared aortic root diameters between the postoperative and the follow-up period. Ro et al 21 determined that a maximal preoperative root diameter >45 mm was an independent predictor of the composite endpoint of aortic regurgitation and root dilatation during follow-up. Assessment of the indexed diameter of the sinus of Valsalva 11 was added in our study, so as not to underestimate the number of patients (despite an aortic root diameter <45 mm) still harbor a substantial aortic root enlargement compared with body size.

New-onset aortic regurgitation ≥grade 2 is more frequent in the SCR group than in ARR group. This incidence may be underestimated in the SCR group because of scarcity of echocardiographic data available during follow-up (57.1% patients). A few studies 21 report incidence from 3 to 4.3% of significant aortic regurgitation during follow-up.

### Limitations

This is a historical monocentric retrospective study of patients who were operated from TAAD and did not consider surgical evolution during the two decades of follow-up techniques incorporated in our practice, especially concerning the growing number of valve-sparing root operations. Follow-up percentages are short of complete, but realistic for a study of this duration.

## Conclusion

TAAD surgery with a conservative approach is an effective strategy in terms of reoperation of the aortic root, although ARR improved overall survival (possibly because the ARR group was much younger).
